# Closed-Loop Control and Output Stability Analysis of a Micromechanical Resonant Accelerometer

**DOI:** 10.3390/mi13081281

**Published:** 2022-08-08

**Authors:** Heng Liu, Yu Zhang, Jiale Wu

**Affiliations:** School of Electronic & Information Engineering, Nanjing University of Information Science & Technology, Nanjing 210044, China

**Keywords:** resonant accelerometer, sensitivity, self-excited oscillation, frequency stability, Allan variance

## Abstract

In this study, a dynamic equation for a micromechanical resonant accelerometer based on electrostatic stiffness is analyzed, and the parameters influencing sensitivity are obtained. The sensitivity can be increased by increasing the detection proof mass and the area facing the detection capacitor plate and by decreasing the stiffness of the fold beams and the initial distance between the plate capacitors. Sensitivity is also related to the detection voltage: the larger the detection voltage, the greater the sensitivity. The dynamic equation of the closed-loop self-excited drive of the accelerometer is established, and the steady-state equilibrium point of the vibration amplitude and the stability condition are obtained using the average period method. Under the constraint conditions of the PI controller, when the loading acceleration changes, the vibration amplitude is related to the reference voltage and the pre-conversion coefficient of the interface circuit and has nothing to do with the quality factor. When the loading voltage is 2 V, the sensitivity is 321 Hz/g. Three Allan variance analysis methods are used to obtain the frequency deviation of 0.04 Hz and the amplitude deviation of 0.06 mVwithin 30 min at room temperature. When the temperature error in the incubator is ±0.01 °C, the frequency deviation decreases to 0.02 Hz, and the resolution is 56ug. The fully overlapping Allan variance analysis method (FOAV) requires a large amount of data and takes a long time to implement but has the most accurate stabilityof the three methods.

## 1. Introduction

The advantages of micromechanical resonant accelerometers are their small size, low power consumption, mass production, and their use of strong anti-interference output as a frequency signal. They are widely used to measure acceleration, displacement, force, etc. The working principle of this technology is to change the stiffness of the resonant beam through acceleration to change the resonant frequency [1]. There are two main types of stiffness changes: inherent stiffness change types and external electrostatic negative stiffness types. With the intrinsic stiffness change type, acceleration acts on the movable mass to generate an inertial force; the inertial force directly loads onto the axial end of the vibrating beam through a lever [2,3]. In order to improve the sensitivity and common mode rejection ratio (CMRR) of axial force-sensitive resonant accelerometers, two identical micromechanical levers are generally used. However, manufacturing process errors will lead to different force coefficients and nonlinearity of sensitivity. When the micromechanical lever amplifies the axial force, it also increases the coupling sensitivity of the cross axis [4,5]. At the same time, the levers need long-term repetitive work, and there is a risk of fatigue fracture.Once the resonant frequency is determined, there is deviation between the resonant frequency and the expected design value due to manufacturing errors. As such, subsequent adjustments to the sensitivity and other parameters for inherent stiffness change-type resonant accelerometers cannot be realized [6].

When a voltage is loaded to the movable capacitor plate, the movable plate produces an equivalent electrostatic negativestiffness, and its magnitude is related to the loading voltage, the area facing the plates, the distance between the plates, and the dielectric constant of the medium between the plates. The equivalent stiffness of the movable plate decreases due to electrostatic negative stiffness, so the resonance frequency of the movable plate also decreases [7,8,9]. Micromechanical resonant accelerometers that are based on external electrostatic negative stiffness are able to tune the sensitivity according to the loading voltage, and the sensitivity has little dependence on manufacturing errors [6]. There are two typical forms of electrostatic stiffness resonant accelerometers: in the first type, the plate distance changes through acceleration [10,11,12,13,14], and for the other, the area facing the plate is changed through acceleration [15,16,17]. The former is in-plane vibration, and the latter is out-of-plane vibration. The two-mode shapes are different, and the last is much more nonlinear.

Both axial forces sensitive and electrostatic stiffness types have two movable parts, one is the resonant beam, which is responsible for the output frequency to characterize the acceleration, and the other is the sensitive mass block subsystem, which converts the acceleration into the force loaded on the axial direction of the resonant beam. The motion directions of the two movable parts are orthogonal, and the motion of the sensitive mass subsystem does not affect the motion displacement of the resonant beam so that the sensitivity can be easily calculated by the resonant beam [2,3]. However, for the electrostatic stiffness accelerometer, the two moving parts are coupled in the same direction. Especially for the in-plane moving electrostatic stiffness resonant accelerometer, the displacement of the resonant beam is affected by the displacement of the mass subsystem, so it is difficult to give the sensitivity relationship directly [10,11,12,13,14]. The sensitivity is obtained through experimental tests, and it is difficult to guide optimal microstructure design. The sensitivity analysis of the electrostatic stiffness resonant accelerometer with in-plane vibration has not been investigated. The analytical expression of sensitivity is helpful for better design and optimization of the layout of microstructure, so this part of the work has a clear significance.

When micromechanical resonant accelerometers are in the resonance state, the amplitude of the detection signal and the signal-to-noise ratio at the detection end corresponding to the same amplitude of the driving force are the largest. At the same time, to reduce cross-coupling between the vibration amplitude and the resonant frequency, it is necessary to maintain the constant amplitude vibration of the vibrating beam [18]. Automatic gain control is widely used in continuous amplitude control; phase-locked loops and self-excited oscillation are often used to achieve frequency tracking control [19,20,21]. The authors of reference [20] analyzed the closed-loop control theory under automatic gain and used a phase-locked loop using the average period method; however, they did not investigate or experimentally verify frequency stability and amplitude stability using the control method, affecting how the resolution was tested and analyzed. Allan variance is often used to analyze the phase noise of resonant sensors. During analysis, the sampling sequence is first grouped using the cluster analysis method, and the mean value of each group is used as the subsequent analysis target [22]. According to different grouping methods, Allan variance is divided into fully overlapping Allan variance (FOAV), incomplete overlapping Allan variance (NFOAV), and non-overlapping Allan variance (NAV) [23]. Different variance analysis methods have different application scenarios for different sample sizes, analysis times, and precision requirements.

This study focuses on three issues: the first issue aims to solve the relationship between the sensitivity of the electrostatic stiffness of the resonant micro accelerometer and the structural parameters as well as the loading voltage to guide the layout optimization design of the accelerometer; the second is to determine whether the steady-state behavior of the closed-loop self-excited system meets the amplitude and frequency requirements based on the dynamic behavior analysis; and the third is to analyze the stability of the resonance frequency and vibration amplitude under closed-loop self-excitation to determine the resolution and stability of the accelerometer.

## 2. Working Principles of Micromechanical Resonant Accelerometer

**Figure 1:**
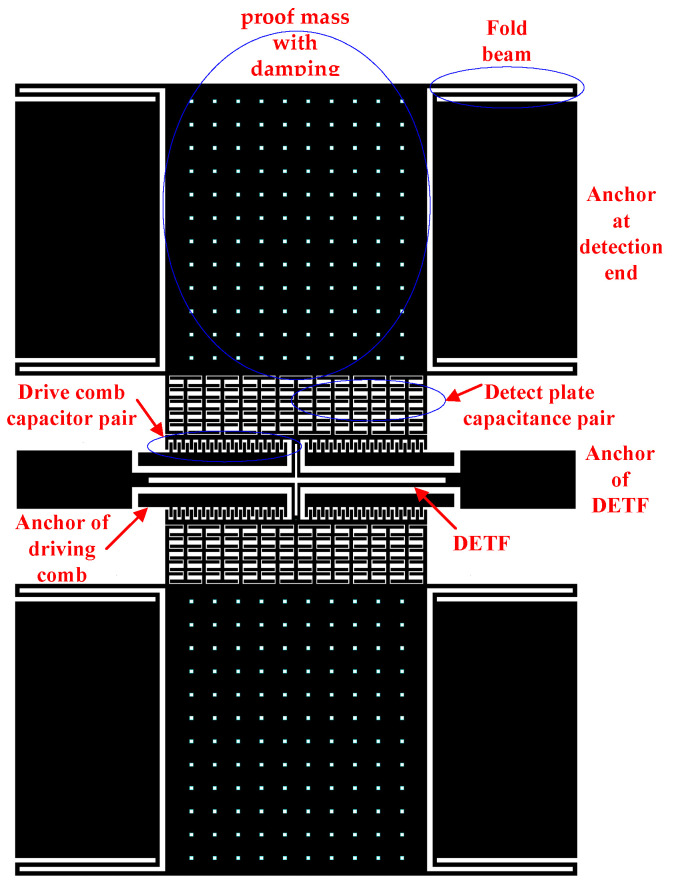
Resonant micro-accelerometer.

The structural layer of the resonant micro-accelerometer includes a sensitive proof mass with some damping holes, the folded beams supporting the suspended proof mass, the detection plate capacitor pair attached to the proof mass, the fixed driving comb capacitor pair, a double-ended tuning fork (DETF) resonant beam, and fixed anchors, as shown in Figure 1. The Y-axis is the drive and detection direction. By adopting single-side drive and single-end detection, the structure of the accelerometer can be divided into two identical single-beam resonant accelerometers at the middle symmetry point. The folded beam and the proof mass structure are connected to the detection voltage $V_{s}$, the movable tuning fork beam is connected to the high-frequency square wave $V_{a}$, and the fixed comb is connected to the AC voltage $V_{ac}\sin\omega t$ and the DC bias voltage $V_{d}$. The resonant beam structure is equivalent to a second-order system, and the input–output relationship is analogous as a band-pass filter. For the low-amplitude high-frequency square wave voltage $V_{a}$, the frequency is far from the resonant frequency and is equivalent to grounding. When the DC amplitude of the driving voltage $V_{d}$ is much larger than the AC amplitude $V_{ac}$, and acceleration a is applied, and the output frequency of the single resonant beam $f_{e}$ can be expressed as

(1) $$f_{e}=\frac{1}{2\pi }\sqrt{\frac{k_{m}-k_{e}(a)}{m}}$$

In Formula (1), $k_{m}$ is the stiffness of the tuning fork beam, m is the mass of the tuning fork beam and its attached microstructure, and $k_{e}(a)$ is the electrostatic stiffness when there is acceleration, which is related to a. The proof mass and the plate at the detection end will be displaced by Δd:

(2) $$\Delta d=\frac{m_{s}\cdot a}{k_{s}}$$

In Formula (2), $m_{s}$ represents the mass of the proof mass with damping holes and its attached microstructure, $k_{s}$ is the equivalent stiffness of the four folded beams, and the corresponding electrostatic stiffness $k_{e}(a)$ is

(3) $$k_{e}(a)=\frac{\varepsilon \cdot S\cdot V_{s}{}^{2}}{{\left(d_{0}-\Delta d\right)}^{3}}=\frac{\varepsilon \cdot S\cdot V_{s}{}^{2}\cdot k_{s}{}^{3}}{{\left(d_{0}\cdot k_{s}-m_{s}\cdot a\right)}^{3}}$$

where ε is the dielectric constant of air and S is the area facing the detection capacitor plate. The structural design requirements are $k_{s}\ll k_{m}$ and $\Delta d\ll d_{0}$, resulting in

(4) $$f_{e}\approx f_{0}(1-\frac{3\beta }{2}\frac{\Delta d}{d_{0}}-(3\alpha +\frac{9}{8}\alpha^{2}){\left(\frac{\Delta d}{d_{0}}\right)}^{2}+o{\left((\frac{\Delta d}{d_{0}})\right)}^{2}\cdots )$$

where

$$\beta =\frac{\varepsilon \cdot S\cdot V_{s}{}^{2}}{d_{0}{}^{3}\cdot k_{m}},\;\alpha =\frac{\varepsilon \cdot S\cdot V_{s}{}^{2}}{d_{0}{}^{3}\cdot k_{m}}\cdot (1-\frac{\varepsilon \cdot S\cdot V_{s}{}^{2}}{d_{0}{}^{3}\cdot k_{m}}),\;f_{0}=\frac{1}{2\pi }\sqrt{k_{m}/m}$$

Ignoring higher-order terms, $f_{e}$ can be expressed as

(5) $$f_{e}\approx \frac{1}{2\pi }\sqrt{k_{m}/m}\cdot (1-\frac{3\varepsilon \cdot S\cdot V_{s}{}^{2}}{2d_{0}{}^{3}\cdot k_{m}}\frac{m_{s}\cdot a}{d_{0}\cdot k_{s}})$$

According to Formula (5), the resonant frequency $f_{e}$ has an approximately linear relationship with the acceleration a. The sensitivity can be increased by increasing the proof mass $m_{s}$ and the area facing the detection capacitor plate S and by decreasing the equivalent stiffness $k_{s}$ and the initial distance between the plate capacitors $d_{o}$. The sensitivity is also related to the square of the detection voltage $V_{s}$; the larger $V_{s}$ is, the greater the sensitivity will be.

**Figure 2:**
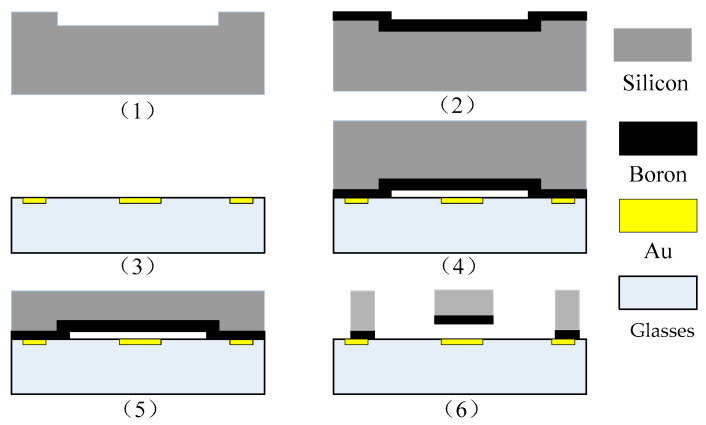
Bulk silicon technological process. (**1**) clean the monocrystalline silicon wafer and etch the bonding platform; (**2**) dope with concentrated boron via a diffusion process; (**3**) sputter Au on the borosilicate glass; (**4**) bond the glass and silicon through the anode; (**5**) dry-etched with excess silicon; (**6**) etch the back of the silicon wafer.

**Figure 3:**
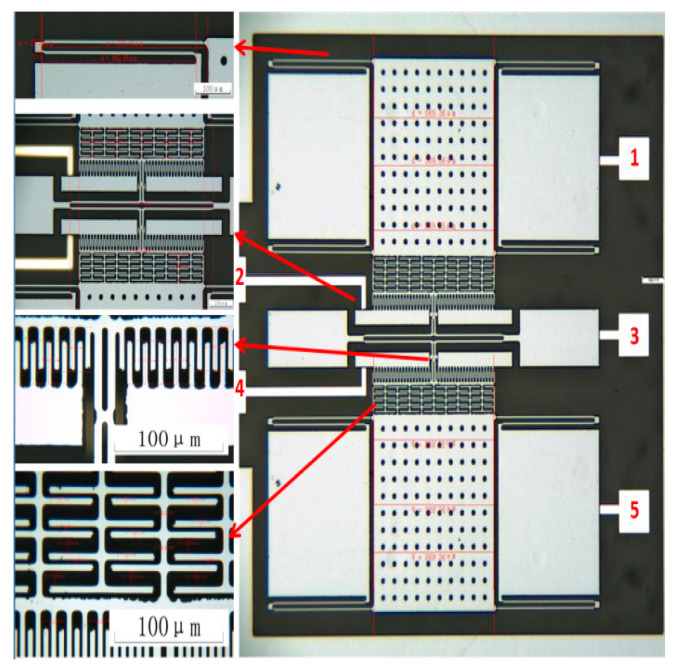
Fabricated accelerometer.1. the upper electrode connected to sensing voltage; 2. the upper electrode connected to driving voltage; 3. the electrode connected to the tuning fork beam; 4. the lower electrode connected to driving voltage; 5. the lower electrode connected to sensing voltage.

The structural layer of the accelerometer was made of a monocrystalline silicon material doped with concentrated boron to improve the conductivity of the microstructure. Pyrex 7740 glass was used as the substrate material, and this material anode bonded the microstructure and the substrate. Inductively coupled plasma (ICP) etching technology was used to obtain larger depth and width ratios. In step 1, the monocrystalline silicon wafer was cleaned, and the bonding platform was etched; in step 2, the monocrystalline silicon was doped with concentrated boron via a diffusion process to increase the conductivity of the structure; in step 3, Au was sputtered on the borosilicate glass, followed by the photolithography electrode and lead; in step 4, the glass and silicon were bonded through the anode; in step 5, the back of the bonded silicon wafer was dry-etched with excess silicon, and the structural layer was thinned; in step 6, the back of the silicon wafer was etched with the deep silicon etching process. The process flow is relatively simple and has high yield; the gap between the structure and the substrate is easy to control; there are fewer pollution impurities, only three masks, and three photolithography periods; and the process is inexpensive. The process flow is shown in Figure 2. The fabricated accelerometer is shown in Figure 3, and a computer vision method was used to directly mark the length and width dimensions. Table 1 shows the structural parameters that were designed and measured.

**Figure 4:**
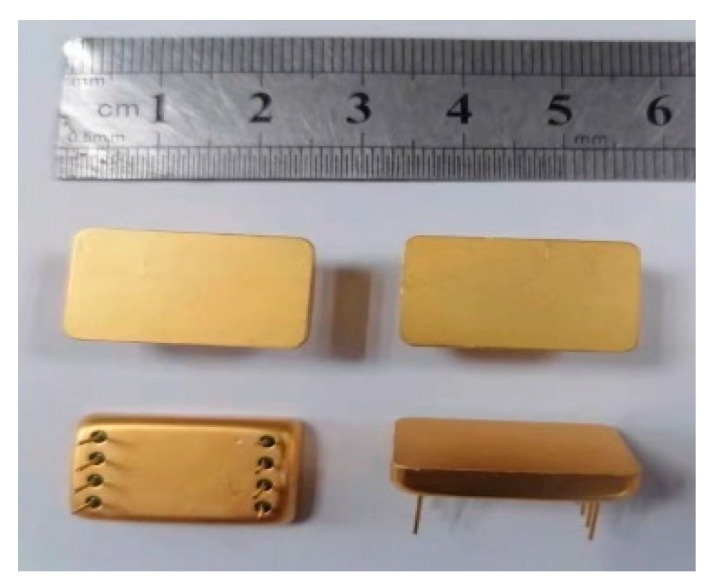
Vacuum-packaged accelerometer.

A WykoNt1100 optical profiler (VeecoInstruments Inc., Plainview, NY, USA) was used to test the accelerometer under an atmosphericpressure package. The test showed that the vibration amplitude was less than 10 nm and that the interface circuit was difficult to detect. Therefore, the microstructure was vacuum-packaged in a metal tube and shell to reduce resonance energy consumption. First, a single accelerometer chip was cut out of the silicon chip using laser-scribing technology; second, we applied an epoxy resin sealant on the bottom of the metal tube shell and stuck the independent chip on the surface of the substrate; third, using ultrasonic wire-bonding technology, the Au wire on the glass substrate was connected to the metal wire on the metal shell, and the shell was then capped using an ultrasonic welding process in a nitrogen environment; finally, the micro accelerometer was placed in a temperature box and maintained at a high temperature of 60 °C for two hours and then reduced to room temperature to complete the packaging of the whole device. The packaged accelerometer is shown in Figure 4 and uses a dual in-line package with eight pins (20.8 mm × 12.7 mm × 5.5 mm). The degree of vacuum was estimated at nearly 30mTorr and was maintained under high-vacuum conditions using a getter within the package.

## 3. Design of Measurement and Control Circuit for the Accelerometer

**Figure 5:**
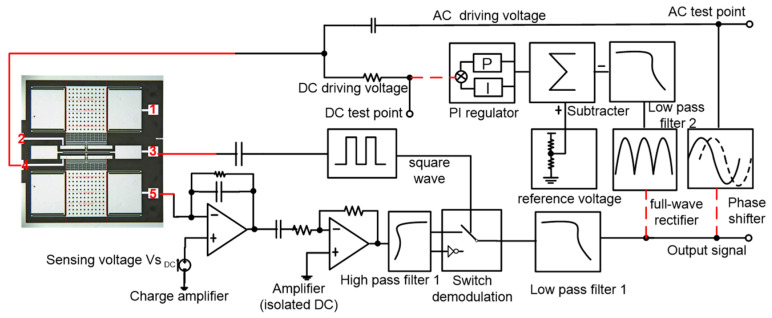
The control circuit of the micro-accelerometer. 1. the upper electrode connected to sensing voltage; 2. the upper electrode connected to driving voltage; 3. the electrode connected to the tuning fork beam; 4. the lower electrode connected to driving voltage; 5. the lower electrode connected to sensing voltage.

The fabricated micromechanical resonant accelerometer is symmetrical up and down and can be divided into two identical single resonant beam accelerometers. Taking a single resonant beam as an example, the measurement and control circuit includes a charge amplifier, an AC amplifier, a high-pass filter, a square wave generation circuit, a switch demodulation circuit, low-pass filter 1, a full-wave rectifier, low-pass filter 2, a phase shifter, a voltage divider, a subtraction circuit, a proportional-integral adjustment circuit, and a power supply circuit, as shown in Figure 5. Detection capacitance exists between detection electrode 5 and tuning fork electrode 3, and a fixed capacitance also exists between detection electrode 5 and driving electrode 4. Detection electrode 5 is connected to the charge amplifier, and the output signal has co-frequency interference, which is mainly generated by the coupling capacitance between detection electrode 5 and driving electrode 4. To eliminate co-frequency interference, the interface circuit adopts high-frequency square-wave modulation and switches the demodulation circuit [24].

**Figure 6:**
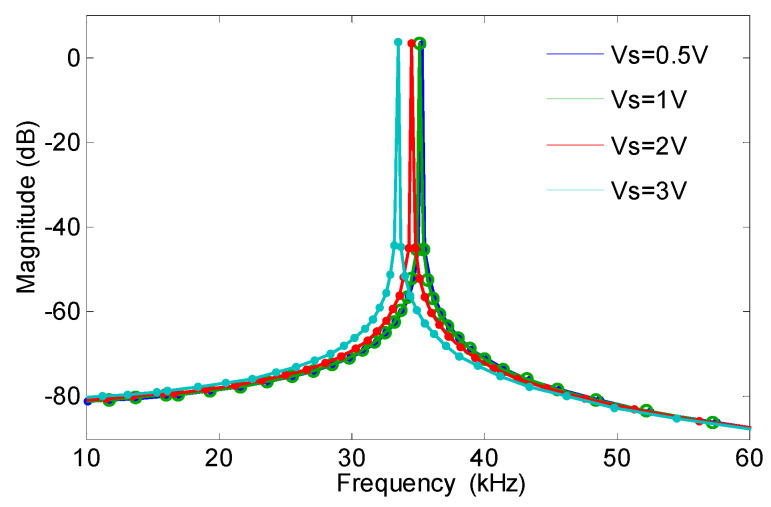
Amplitude-frequency curve of micro-accelerometer sweep frequency test.

During the open-loop test, the acceleration in the sensitive direction was 0 g, and the three red-dotted circuits were disconnected, as shown in Figure 5. The AC test point was obtained by the AC drive voltage and was determined using the Agilent35670A dynamic signal analyzer (Agilent Technologies Inc., Santa Clara, CA, USA). The DC test point was determined using the DC drive voltage via the DC-stabilized power supply, and the signal output from the low-pass filter was connected to the feedback input of the Agilent35670A. The amplitude-frequency curve was obtained when the sweep frequency range was 10–60 kHz, as shown in Figure 6. As the detection voltage was 1 V, the corresponding resonance frequency was 35.2986 kHz, and the quality factor Q was 1076. The corresponding resonant frequency changed nonlinearly with the change in the detection voltage. 

In the closed-loop measurements and control circuit design, the output sine wave signal was obtained through low-pass filter 1. One channel of the sine wave signal was connected to the driving comb electrode through the all-pass phase shifter. The other channel obtained the amplitude of the sine wave through full-wave rectification and low-pass filter 2. The DC amplitude voltage was subtracted from the reference voltage and was connected to the driving comb electrode through a resistor after proportional-integral adjustment, which resulted in automatic AC-DC control [25].

**Figure 7:**
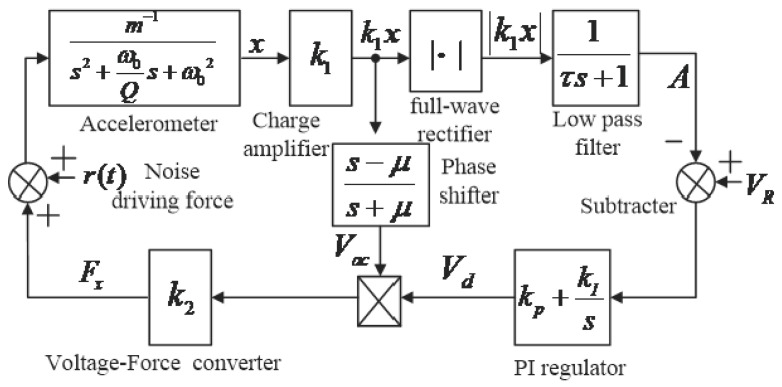
The closed-loop measurement and control circuit.

The sinusoidal signal, which was obtained from the open-loop test was proportional to the vibration displacement of the microstructure. Combined with Figure 5, the corresponding dynamic diagram is shown in Figure 7 and included two loops. One loop maintains the phase balance [26], and the other keeps the vibration amplitude constant. In Figure 7, $k_{1}$ is the gain coefficient of the charge amplifier; τ is the time constant of low-pass filter 2; A is the DC voltage after filtering; $V_{R}$ is the DC reference voltage; $V_{d}$ is the DC driving voltage; $V_{ac}$ is the AC driving voltage; μ is the time constant of the phase shifter; $k_{p}$ and $k_{I}$ are the proportional and integral coefficients, respectively; $k_{2}$ is the voltage-force conversion coefficient related to the driving comb; and r(t) is the equivalent driving force generated by thermal noise. Supposing that the vibration displacement of the resonant beam is x(t), $x(t)=a(t)\cos(\omega_{d}t+\phi (t))$, the amplitude is a(t), the phase is ϕ(t), and the angular frequency is $\omega_{d}$.

According to the dynamic principle of each module, the following analysis model was established:

(6) $$\left\{ \begin{array}{l} m(\ddot{x}+\frac{\omega }{Q}\dot{x}+\omega^{2}x)=-K\cdot V_{d}a(t)\sin(\omega_{d}t+\phi (t)+\varphi )\\ \dot{A}(t)=\frac{1}{\tau }(\left|k_{1}x\right|-A(t))\\ {\dot{V}}_{d}=k_{p}({\dot{V}}_{R}-\dot{A})+k_{I}(V_{R}-A) \end{array}\right.$$

where $K=k_{1}\cdot k_{2}$ and $\ddot{x}(t)$, $\dot{x}(t)$, and x(t) are substituted into formula system (6), and the slow time-varying system is solved with the principle of the averaging method as follows:

(7) $$\dot{a}(t)=-\frac{1}{2}a(t)\cdot (\frac{\omega_{d}}{Q}-\frac{KV_{d}}{m\omega_{d}}\cos\varphi )$$

(8) $$\dot{\phi }(t)=\frac{1}{2}\frac{KV_{d}}{m\omega_{d}}\sin\varphi$$

(9) $$\dot{A}(t)=\frac{1}{\tau }(\frac{2}{\pi }k_{1}a(t)-A(t))$$

(10) $${\dot{V}}_{d}(t)=k_{I}(V_{R}-A(t))-k_{p}\frac{1}{\tau }(\frac{2}{\pi }k_{1}a(t)-A(t))$$

When the quality factor is large, the change in state variables of the slow time-varying system is close to 0. By making each state variable be 0 after derivation with time, two equilibrium points are obtained as follows:

(11) $$\left\{ \begin{array}{l} \bar{a_{0}}=0\\ \bar{A_{0}}=0 \end{array}\right.$$

(12) $$\left\{ \begin{array}{l} \bar{a_{1}}=\frac{\pi V_{R}}{2k_{1}}\\ \bar{A_{1}}=V_{R}\\ \bar{V_{1d}}=\frac{m\omega_{d}{}^{2}}{KQ\cos\varphi } \end{array}\right.$$

The variables a(t), A(t), and $V_{d}(t)$ represent linearization at the equilibrium points $\left(\bar{a_{1}},\bar{A_{1}},\bar{V_{1d}}\right)$, and the corresponding state equations are:

(13) $$\dot{a}(t)=-\frac{\omega_{d}}{2Q}(a(t)-\frac{\pi V_{R}}{2k_{1}})+\frac{\pi V_{R}}{4k_{1}}(V_{d}-\frac{\omega_{d}}{Q})$$

(14) $$\dot{A}(t)=\frac{1}{\tau }\frac{2}{\pi }k_{1}(a(t)-\frac{\pi V_{R}}{2k_{1}})-\frac{1}{\tau }(A(t)-V_{R})$$

(15) $${\dot{V}}_{d}(t)=(k_{p}\frac{1}{\tau }-k_{I})(A(t)-V_{R})-k_{p}\frac{1}{\tau }\frac{2}{\pi }k_{1}(a(t)-\frac{\pi V_{R}}{2k_{1}})$$

The state equation characteristic matrix is:

$$\left|\begin{array}{ccc} \begin{array}{l} \frac{\omega_{d}}{2Q}\\ \frac{2k_{1}}{\tau \pi }\\ \frac{2k_{1}k_{p}}{\tau \pi } \end{array} & \begin{array}{l} \begin{array}{cc} & 0 \end{array}\\ -\frac{1}{\tau }\\ \frac{k_{p}}{\tau }-k_{I} \end{array} & \begin{array}{l} \frac{\pi V_{R}}{4k_{1}}\\ 0\\ 0 \end{array} \end{array}\right|$$

The eigenvalue of the matrix λ is solved as follows:

(16) $$\lambda^{3}+(\frac{\omega_{d}}{2Q}+\frac{1}{\tau })\lambda^{2}+(\frac{\omega_{d}}{2Q\tau }+\frac{V_{R}k_{p}}{2\tau })\lambda +\frac{V_{R}}{2\tau }(\frac{2k_{p}}{\tau }-k_{I})=0$$

According to the Routh criterion, the vibration stability of the driving mode needs to satisfy the below conditions:

(17) $$k_{I}<\frac{2k_{p}}{\tau }$$

Thesystem has different poles and demonstrates different dynamic performances when changing $k_{p}$, $k_{I}$, and τ in the system. According to Formula (12), the steady-state amplitude a(t) is related to $V_{R}$ and $k_{1}$ and has nothing to do with the microstructure’s stiffness, mass, resonant frequency, or quality factor. Under temperature disturbance, the amplitude of the closed-loop drive mode that corresponds to the frequency drift remains unchanged, and the measurement and control circuit can realize constant amplitude vibration and frequency tracking.

The system has two equilibrium points. When the amplitude of the equilibrium point $\left(\bar{a_{0}},\bar{A_{0}}\right)$ is 0, the microstructure does not start to vibrate, and it is an unstable equilibrium point. If the microstructure can start to vibrate, the coefficient of the first-order term of the linear equation is greater than 0 ((a(t)>0)), which satisfies the following equation:

(18) $$-\frac{1}{2}(\frac{\omega_{0}}{Q}-\frac{KV_{R}}{m\omega_{0}}\cos\varphi )>0$$

After solving inequality (18), we achieve

(19) $$V_{R}>\frac{m\omega_{0}{}^{2}}{KQ\cos\varphi }$$

According to Formula (19), $V_{R}$ must be greater than the DC driving voltage corresponding to the static equilibrium point so that the microstructure of the driving mode can start to vibrate stably. The phase shift error φ should be 0 under ideal conditions. When there is a phase offset, the larger the deviation angle, a larger $V_{R}$ is required [26].

**Figure 8:**
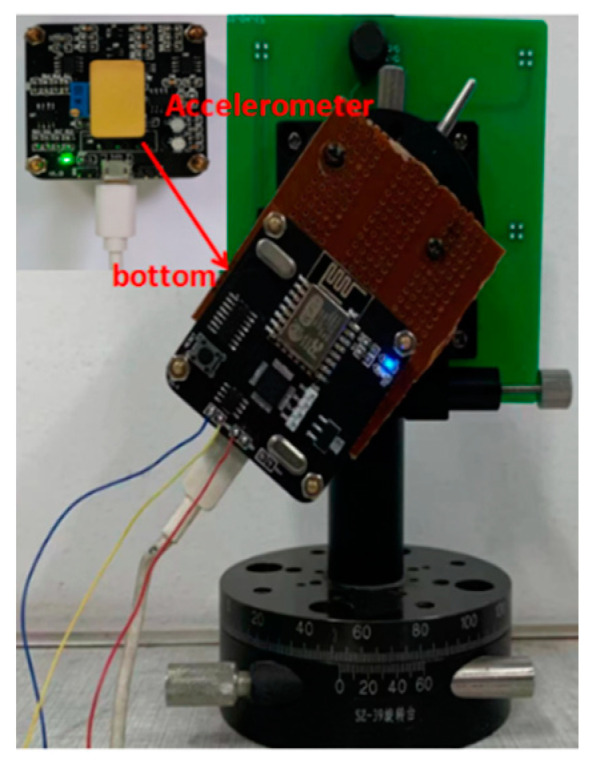
Experimental device of the circuit.

**Figure 9:**
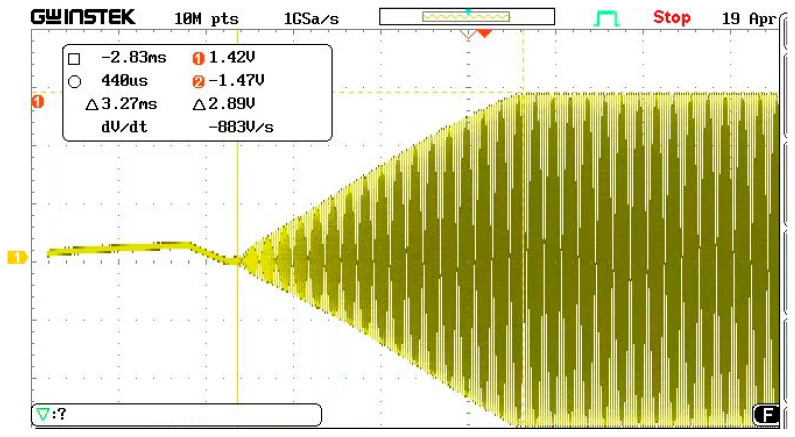
Oscillation observation waveform.

According to the theoretical analysis, a self-excited oscillation measurement and control circuit is established, as shown in Figure 8. The measurement and control circuit are on the left, and the phase shifter adjusts the phase through a rheostat. When the acceleration in the sensitive direction is 0 g and the detection voltage is 1 V, the oscillation amplitude gradually increases and becomes stable after the micro-accelerometer is powered on. After adjusting the automatic gain circuit, the oscillation amplitude is constant. The vibration waveform is shown in Figure 9. After 2.83 ms, the amplitude was stable, and the peak-to-peak value was 2.89 V.

After partially enlarging the waveform shown in Figure 9, the wave was a symmetrical sine wave, which is consistent with the theoretical design. The spectrum analyzer was used to analyze the stabilized sine wave, and the corresponding resonance frequency was 35.300 kHz, and the power was about 2.4 dBV.

**Figure 10:**
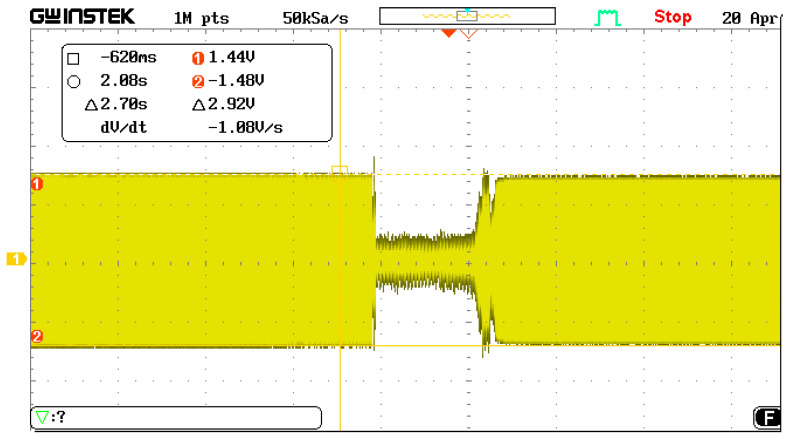
The acceleration jump oscillation process.

The accelerometer was manually placed into a tilted position, the acceleration changed from 0 g to 1 g, and the electrical signal corresponding to the vibration of the resonant beam is shown in Figure 10. When the acceleration jumped, the vibration amplitude was stable. As the acceleration changed, the amplitude was the same, and the middle represents the manual flip time of the accelerometer.

**Figure 11:**
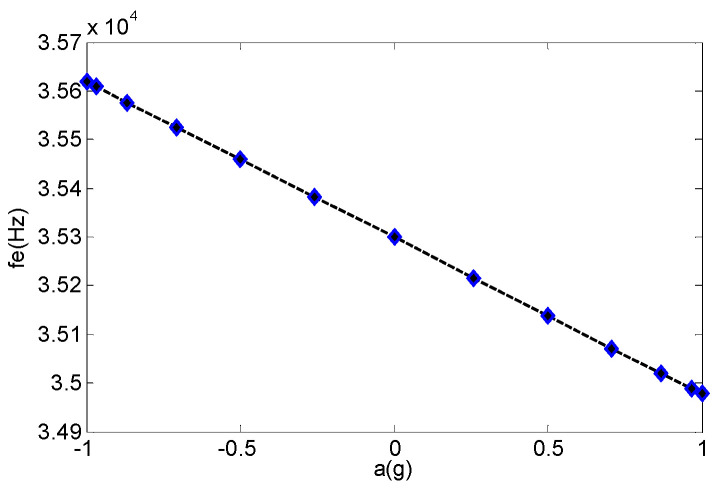
Sensitivity of the micro-accelerometer.

The self-made rotary slide and dial were able to achieve precise adjustments of 0.5° and to provide acceleration input from −1 g to 1 g through gravity decomposition. The test instrument is shown in Figure 8, and the corresponding voltage supply mode remained unchanged. The dial was adjusted to achieve a difference in the rotation angle of 15° and to decompose the acceleration into the corresponding sine components. When the detection voltage changed to 2 V and the corresponding acceleration rotation angle rangedfrom −90° to 90°, the relationship between the acceleration and output frequency is shown as in Figure 11, and the sensitivity was 321 Hz/g. When the detection voltage was 3 V, the large detection voltage corresponded to high sensitivity, but the output frequency and acceleration had a seriously nonlinear relationship.

## 4. Analysis and Experiment of Output Stability

The micromechanical resonant accelerometer is sensitive to acceleration caused by the frequency, and the frequency’s stability determines the resolution [27,28]. At the same time, it has been reported that a large vibration amplitude will lead to the coupling of amplitude and resonant frequency [29], so it is necessary to quickly and accurately analyze the stability of the steady-state vibration amplitude and the resonant frequency [30].

In Allan variance analysis, the sampling sequence is first grouped using the cluster analysis method, and the mean of each group is used as the next analysis target [31,32]. Then, the adjacent cluster means in the mean sequence are differed, and half of the mean square values of all of the differences is the Allan variance value, which represents the error situation of the sample sequence analyzed under a clustering time scale.

The steps for Allan variance analysis are provided below:

1. The sensor output signal is sampled using τ0 as the time interval, and a total of *N* discrete sample data are collected to obtain the sampling sequence χ(i), *i* = 1, 2, 3, …, *N*.

2. Average grouping is performed on the sampling sequence χ(i). First, the length parameter α and step-size parameter β of the sample data group are set. α and β are both positive integers; that is, each group contains α sample data for grouping, and the first data for adjacent groups are separated by the β−1 interval. The time scale of each group is τ = ατ0. Then, parameters α and β are used to group the sampling sequence χ(i), which must satisfy the condition α < *N*/2, and α is an integer multiple of the step-size parameter β. Finally, the average value of each group of data is calculated according to Formula (20), and the mean value sequence γ(j), *j* = 1, 2, 3, …, *K* is obtained. The mean value sample sequence contains *K* data in total, and the exact value of the sequence length *K* can be calculated according to Formula (21):

(20) $$\gamma \left(j\right)=\frac{1}{\alpha }\sum_{i=1}^{\alpha }\chi \left(\left(j-1\right)\beta +i\right)$$

(21) $$K=\left\lfloor \frac{N}{\alpha }\right\rfloor \frac{\alpha }{\beta }-\frac{\alpha }{\beta }+1$$

3. The Allan variance is calculated according to the mean series. First, the difference between two “adjacent” mean samples in the mean sample sequence γ(j) is calculated, and the difference sequence Z(i) is obtained, as shown in Formula (22). Then the mean square value of the difference sequence Z(i) is calculated, and multiplied by 0.5 to determine the Allan variance value, as shown in Formula (23).

(22) $$z\left(i\right)=\gamma \left(i+\frac{\alpha }{\beta }\right)-\gamma \left(i\right)$$

(23) $$\sigma^{2}=\frac{1}{2\left(K-\frac{\alpha }{\beta }\right)}\sum_{i=1}^{K-\frac{\alpha }{\beta }}{z\left(i\right)}^{2}$$

**Figure 12:**
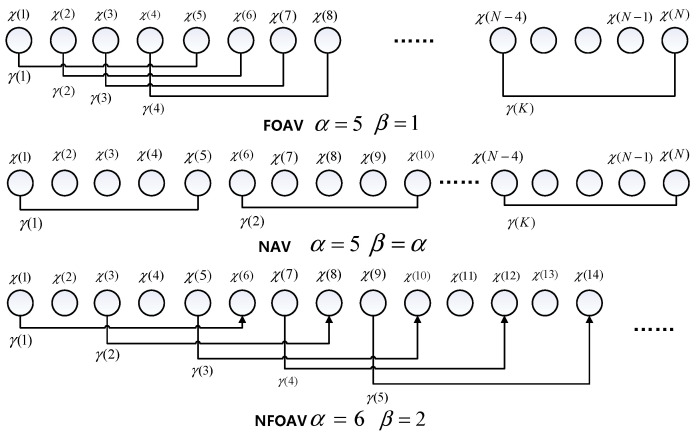
Three Allan variance grouping methods.

Formula (23) is the general calculation formula for Allan variance, and the value range of step parameter β is 1, 2, 3, …, α. Step parameter β = 1 is the fully overlapping Allan variance, β = α is the non-overlapping Allan variance, and 1 < β < α is the incomplete overlapping Allan variance, as shown in Figure 12.

4. The Allan variance is calculated by changing the group length α, and a logarithmic plot is created. Different group length parameters α correspond to different grouping methods and mean sample sequences for the same sampling sequence χ(i), and the Allan variance values calculated by different α parameters are different. The value of parameter α needs to satisfy Formula (21). According to Formula (24), all of the α parameters that meet the requirements are calculated, and the corresponding Allan variance value is obtained. Finally, the time parameter τ = *m*τ0 is used as the independent variable, and the Allan-squared variance is used as the dependent variable to draw a double logarithmic graph. The error and stability of the sampling sequence can be seen in the graph.

(24) $$\alpha <\frac{N}{2},\frac{\alpha }{\beta }=p\left(p=1,2,3\ldots \ldots \right)$$

**Figure 13:**
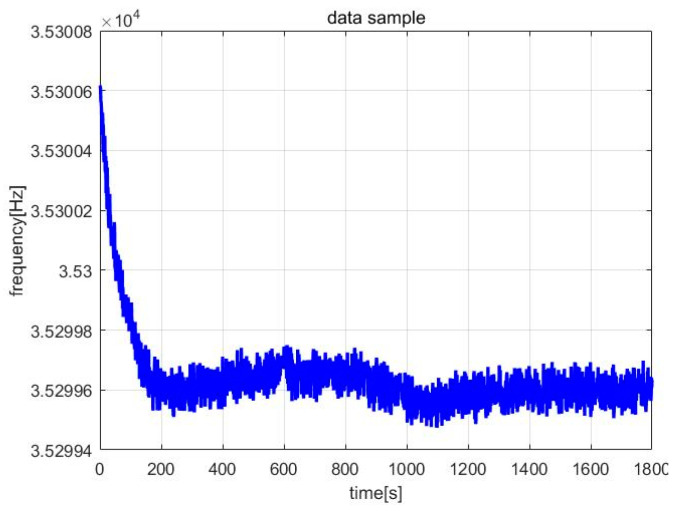
Frequency data samples.

**Figure 14:**
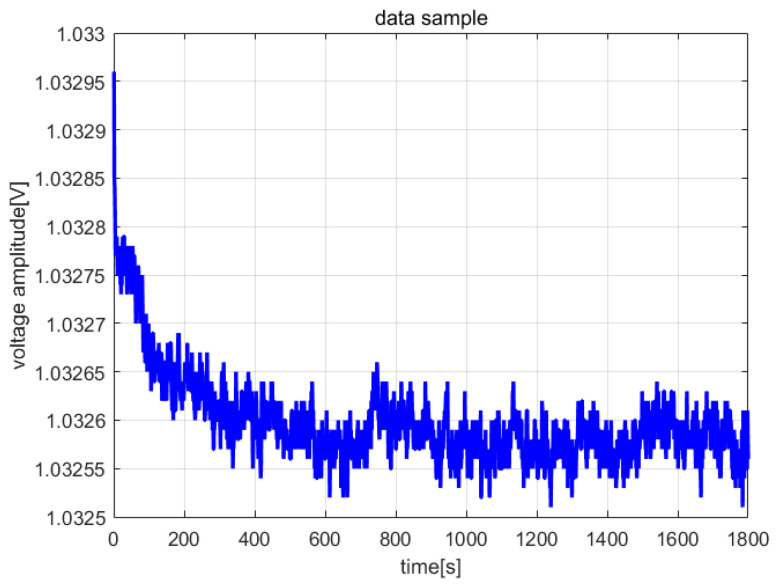
Amplitude data samples.

The frequency meter (Agilent53132A, Agilent Technologies Inc., Santa Clara, CA, USA) and digital multi-meter (DM3068, Beijing RIGOL Inc. Beijing, China) were used to collect the frequency and amplitude data of the steady-state vibration, respectively. The sampling interval was set to 1 s, and the sampling length was 1800. The entire sampling process lasted 30 min. Figure 13 and Figure 14 are the frequency and amplitude data curves, respectively (Vs = 2 V, acceleration is 0 g, room temperature is 28.10 °C).

**Figure 15:**
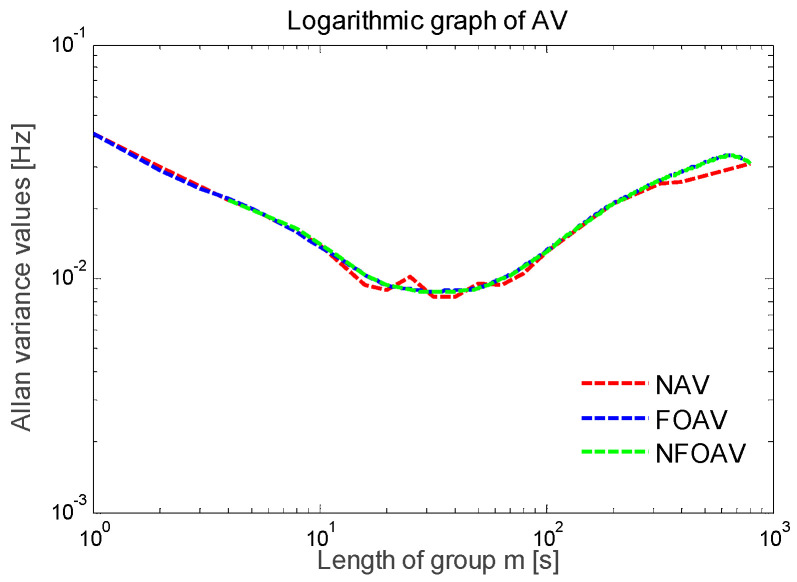
Frequency data curve of Allan variance.

First, the high-precision variance analysis method in MATLAB software (MathWorks Inc. Natick, MA, USA) was used. When the fixed step parameter β = 1 and the group length parameter α traverses from 1 to 900, the Allan variance value is calculated under different group length parameters α. As shown in Figure 15, the curve of stability the analysis results is smooth and stable overall.

Then, the balanced variance analysis method was used. The step parameter is set to β = 2, and the group length parameter α satisfies Formula (24). As shown in Figure 15, the fluctuation of the stability analysis curve was larger than that of the high-precision variance analysis curve.

Finally, the fast analysis of variance method was used. The step parameter is set to β = α, and the group length parameter α traverses all of the factors along the total length of the data *N*. As shown in Figure 15, the curve demonstrating the stability results had many inflection points.

According to Figure 15, consistent curve trends were found in the double logarithmic plots obtained using the three methods. Among them, the high-precision variance analysis curve was the smoothest, and the calculated frequency deviation was the most accurate. The fast analysis variance curve contained the most inflection points and the lowest number of data points. The precision of the balanced variance analysis curve was between the two. The frequency deviation in the steady-state vibration of the accelerometer was less than 0.04 Hz, the resonant frequency was 35.29961 kHz, and the frequency deviation was within ±10 ppm.

**Figure 16:**
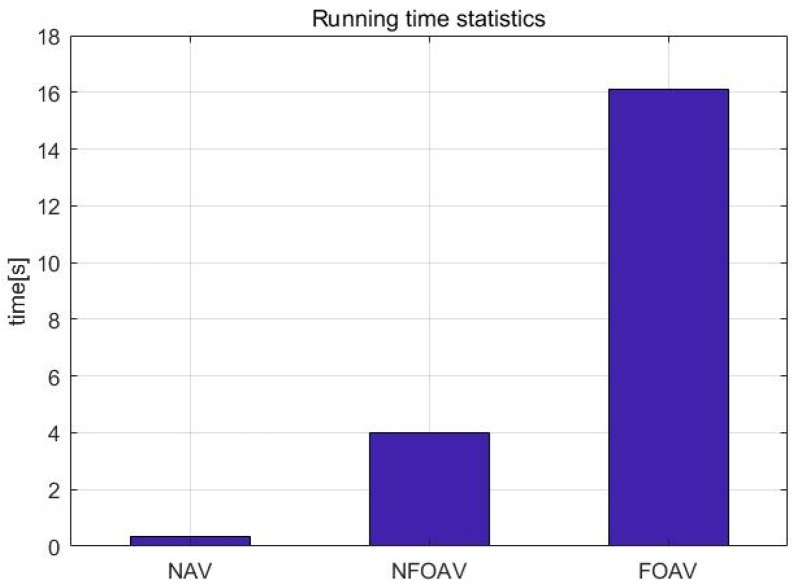
Comparison chart of execution time.

Comparing the running times of the three analysis methods, Figure 16 shows that the execution time of high-precision variance analysis was much longer than that of the other two. The execution time increased exponentially as the length of the frequency sample sequence increased, while the execution time required for fast variance analysis was extremely short, while that required for balanced variance analysis was moderate.

**Figure 17:**
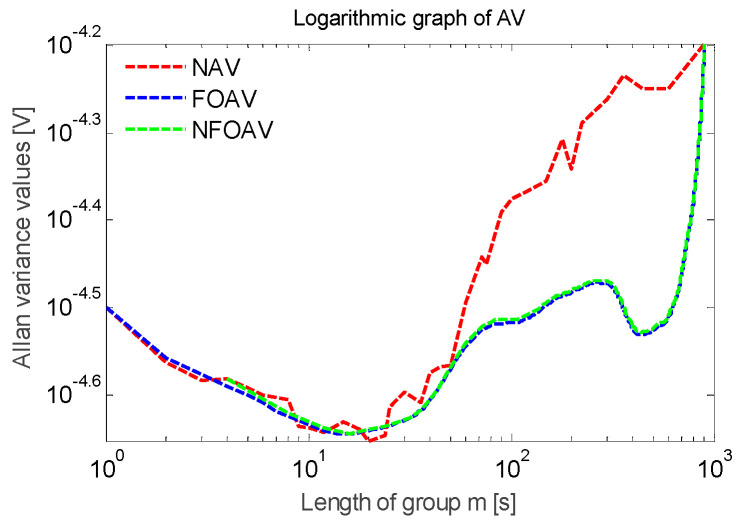
Amplitude data curve for Allan variance.

In the same way, the amplitude data of the steady-state vibration in Figure 14 were used to perform stability analysis. The amplitude of the steady-state vibration signal of the sensor was 2.2 V, and the Allan variance analysis results can be seen in Figure 17. The deviation of the amplitude was less than 0.06 mV and was within ±30 ppm.

**Figure 18:**
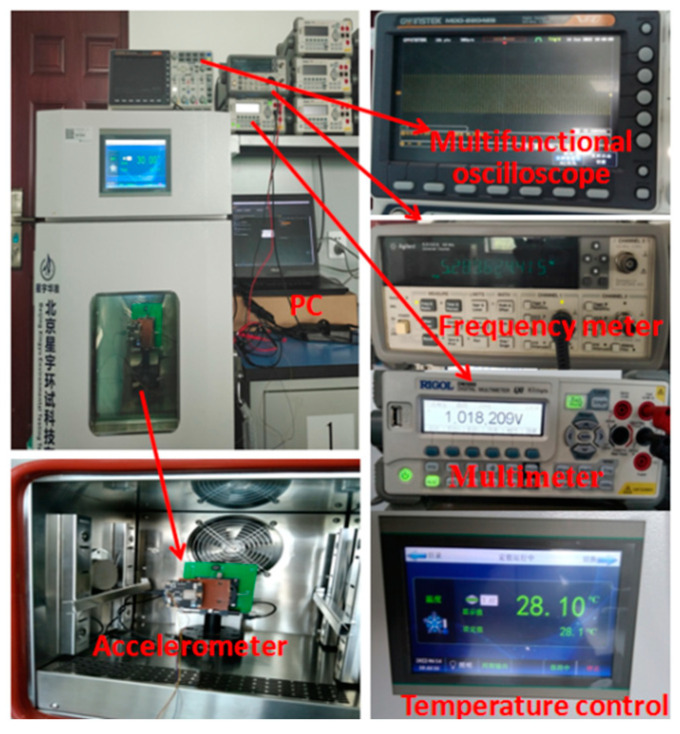
Constant temperature experimental box.

**Figure 19:**
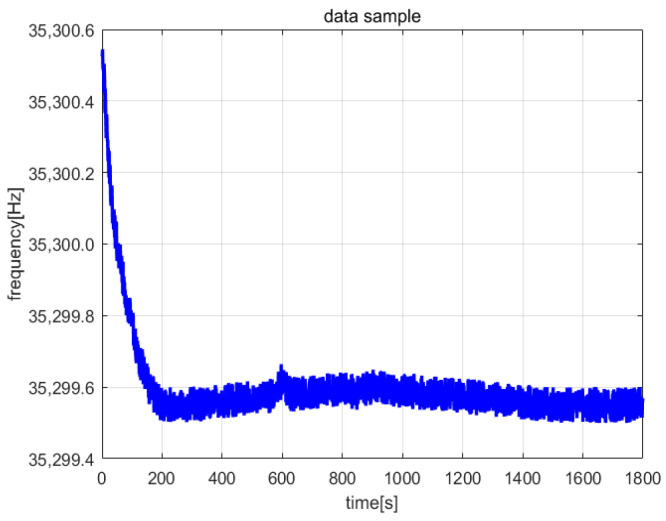
Frequency samples after temperature control.

**Figure 20:**
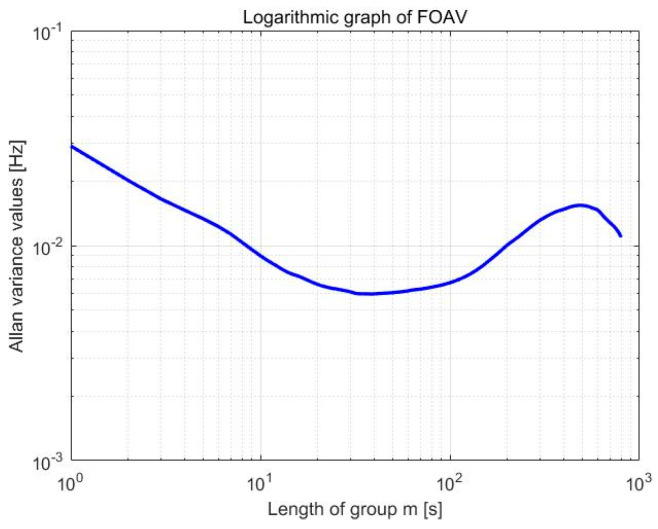
Variance analysis of FOAV.

The constant temperature experiment was carried out using a micromechanical accelerometer. The temperature error of the temperature experimental box was ±0.01 °C, and it was adjusted to 28.10 °C. The experimental device is shown in Figure 18. A frequency meter was used to obtain the frequency data of the steady-state vibration, as shown in Figure 19, and to perform frequency stability analysis, as shown in Figure 20. The frequency deviation was less than 0.02 Hz after temperature control was implemented, which is significantly reduced when compared to that without temperature control. The resolution referring to the sensitivity analysis was determined to be 56 ug. Therefore, ambient temperature significantly affects the frequency stability of the accelerometer, and the resolution is higher under constant temperature conditions.

## 5. Conclusions

This paper designed an in-plane vibration-type resonant micromechanical accelerometer based on electrostatic stiffness. The working principle of the accelerometer was analyzed, and the expression of the sensitivity was deduced. The open-loop experiment confirmed the adjustment effect of the loading voltage on the sensitivity. A large loading voltage corresponded to significant sensitivity, but excessive loading voltage increased nonlinearity. The self-excited oscillation measurement and control circuit experiment based on the average period method showed the vibration amplitude was related to the reference voltage and the preconversion coefficient of the interface circuit and had nothing to do with the quality factor.The stability analysis results of the frequency and vibration amplitude within 30 min at room temperature by the three Allan variance analysis methods were compared. The frequency deviation was determined to be 0.04 Hz, and the amplitude deviation was determined to be 0.06 mV. In the constant temperature experimental box, the temperature was stable within the deviation range of 0.01 °C; the frequency deviation decreased to 0.02 Hz. In Table 2, a comparison is shown between the proposed device and a few resonant accelerometers based on electrostatic stiffness reported in literature. Our work achieved the maximum sensitivity of 321 Hz/g in the range of −1 g to 1 g and the resolution was 56 ug.

Among the three Allan variance analysis methods, high-precision Allan variance analysis has high calculation accuracy but require a large amount of time for implementation; fast Allan variance analysis requires a very short amount of time, but the calculation accuracy is average, and balanced Allan variance analysis can change the balance between its calculation accuracy and execution time.

The axial force-sensitive resonant accelerometer and the electrostatic stiffness resonant accelerometer in this study belong to in-plane vibration. There are both movable resonant beams and acceleration-sensitive mass blocks. However, the motion directions of the resonant beams and acceleration-sensitive mass blocks in the axial force-sensitive accelerometer are perpendicular to each other, so the axial coupling effect is small. The resonant beam and the acceleration-sensitive mass in the electrostatic stiffness resonant accelerometer have the same motion direction and are coupled, and the sensitivity is nonlinear. The plate pull-in effect in the electrostatic stiffness resonant accelerometer limits the increase in the range, and the sensitivity will show significant nonlinearity with the increase in the range. A large electrostatic negative stiffness will also reduce the stability of the accelerometer. Still, the electrostatic stiffness resonant accelerometer has the advantage of using the loading voltage to tune the sensitivity and has little dependence on manufacturing error.

To improve the sensitivity and resolution of the silicon micro resonant accelerometer, the resonant frequency of the silicon micro resonant accelerometer would generally increase significantly, and the layer thickness of the microstructure would be reduced to less than 10um, and the interface capacitance would be weak. The use of special integrated circuits could improve the sensor’s resolution, but it would also increase the technical difficulty and development cost. The measurement and control circuit composed of discrete devices used in the low-frequency resonant accelerometer has a relatively large phase noise and small resolution. In this study, we need to pay attention to the phase noise and the resolution improvement.

## Figures and Tables

**Table 1:** Structural parameters of the accelerometer.

Parameters	Units	Design	Measurement
Length of fold beam	μm	500	472.5
Width of fold beam	μm	8	7.04
Spacing of fold beam	μm	14	16.2
Length of connecting beam	μm	160	148.4
Width of connecting beam	μm	9	7.5
Spacing of connecting beam	μm	4	4.95
Length of drive comb	μm	40	38.5
Width of drive comb	μm	5	4.79
Spacing of drive comb	μm	2	2.5
Pairs of drive comb	pair	19	19
Length of DETF	μm	700	661.6
Width of DETF	μm	8	7.4
Length of detection capacitor	μm	50	45.2
Width of detection capacitor	μm	6	4.87
Spacing of detection capacitor 1	μm	2	2.46
Spacing of detection capacitor 2	μm	10	10.56
Total pairs of detection capacitors		40	40
Length of proof mass	μm	620	609
Width of proof mass	μm	700	683
Length of damping hole	μm	10	12.41
Width of damping hole	μm	10	12.93
Number of damping hole		110	110
Structure layer thickness	μm	40	40.4

**Table 2:** Comparison between this device and reported resonant accelerometers.

Items	Ref. [10]	Ref. [15]	Ref. [16]	Ref. [17]	Ref. [11]	Ref. [12]	Ref. [13]	Ref. [14]	This Work
Year of manufacture	2004	2014	2015	2016	2018	2019	2021	2022	2022
Resonant frequency(kHz)	24.88	24.85	13.69	2.44	3.625	20	186	25.5	35.3
Sensitivity (Hz/g@V)	128@N/A	14@2.5	32@25	10@1.5	5.09@5	20@35	45.8@35	56@15	321@2
Full scale range(g)	±1	±1	±1	0~10	±1	±1	>25	N/A	±1
Resolution (ug)	5.2	N/A	727	N/A	4.3	27	8300	N/A	56
Bandwidth (Hz)	110	N/A	50	100	N/A	N/A	>2000	N/A	>1000
Thickness(um)	40	22	20	22	80	N/A	N/A	60	40

## Data Availability

All data are true and reliable.
